# Empirical Comparison of Visualization Tools for Larger-Scale Network Analysis

**DOI:** 10.1155/2017/1278932

**Published:** 2017-07-18

**Authors:** Georgios A. Pavlopoulos, David Paez-Espino, Nikos C. Kyrpides, Ioannis Iliopoulos

**Affiliations:** ^1^Department of Energy, Joint Genome Institute, Lawrence Berkeley Labs, 2800 Mitchell Drive, Walnut Creek, CA 94598, USA; ^2^Division of Basic Sciences, University of Crete Medical School, Andrea Kalokerinou Street, Heraklion, Greece

## Abstract

Gene expression, signal transduction, protein/chemical interactions, biomedical literature cooccurrences, and other concepts are often captured in biological network representations where nodes represent a certain bioentity and edges the connections between them. While many tools to manipulate, visualize, and interactively explore such networks already exist, only few of them can scale up and follow today's indisputable information growth. In this review, we shortly list a catalog of available network visualization tools and, from a user-experience point of view, we identify four candidate tools suitable for larger-scale network analysis, visualization, and exploration. We comment on their strengths and their weaknesses and empirically discuss their scalability, user friendliness, and postvisualization capabilities.

## 1. Background

Health and natural sciences have become protagonists in the big-data world as high-throughput advances continuously contribute to the exponential growth of data volumes. Nowadays, biological repositories expand every day by hosting various entities such as proteins, genes, drugs, chemicals, ontologies, functions, articles, and the interactions between them, often leading to large-scale networks of thousands or even millions of nodes and connections. As such networks are characterized by different properties and topologies, graph theory comes to play a very important role by providing ways to efficiently store, analyze, and subsequently visualize them [1–5].

Visualization and exploration of biological networks at such scale are a computationally challenging task and many efforts in this direction have failed over the years. Recent review articles [3, 4, 6] discuss the challenges in the biological data visualization field and list a catalog of standalone and web-based visualization tools as well as the visual concepts they are implemented to serve. While these resources are valuable to capture the big picture in the field, get a sense of the available tools, and spot the strengths and the weaknesses of a tool of interest at a glance, no empirical feedback on the tools' scalability was obvious.

To shortly mention representative tools in the field, 2D standalone applications like graphVizdb [7], Ondex [8], Proviz [9], VizANT [10], GUESS [11], UCINET [12], MAPMAN [13], PATIKA [14], Medusa [15], or Osprey [16] as well as 3D visualization tools such as Arena3D [17, 18] and BioLayout Express [19] already exist. Each of them is designed to serve a different purpose. For example, Ondex is implemented to gather and manage data from diverse and heterogeneous datasets, Proviz is dedicated to handle protein-protein interaction datasets, VizANT focuses on metabolic networks and ecosystems, Medusa is able to show semantic networks and multiedged connections, GUESS supports dynamic and time sensitive data, Osprey is implemented to annotate biological networks, Arena3D is targeting multilayered graphs, and BioLayout Express is designed for generic advanced 3D network visualizations.

Despite the fact that such tools are widely used and have great potential for further development, to our experience, they are not recommended for large-scale network analysis in their current versions. UCINET windows application could potentially be used for just visualization purposes. Its absolute maximum network size is about 2 million nodes but, in practice, most of its procedures are too slow to run networks larger than about 5,000 nodes.

Among several existing tools that we tested, we find Cytoscape (v3.5.1) [20], Tulip (v4.10.0) [21], Gephi (v0.9.1) [22], and Pajek (v5.01) [23, 24] standalone applications to be the top four candidates for visualization, manipulation, exploration, and analysis of very big networks. For these four tools, we empirically evaluate their pros and cons, we comment on their scalability, user friendliness, layout speed, offered analyses, profiling, memory efficiency, and visual styles, and we provide tips and advice on which of their features can scale and which of them is better to avoid.

In order to show a representative visualization generated by these four tools, we constructed a graph consisting of 202,424 nodes and 354,468 edges showing the habitat distribution of 202,417 protein families across 7 habitats. Data was collected from the IMG integrated genome and metagenome comparative data analysis system [25] whereas protein families originate from public metagenomes only.

A step-by-step protocol describing how these images were generated is presented as Supplementary Material, available online at https://doi.org/10.1155/2017/1278932. Comments on problems which occurred during our analysis as well as drawbacks and strengths of the visualization tools used for the purposes of this review are extensively discussed.

## 2. Top Four Candidates for Large-Scale Network Visualization

### 2.1. Gephi (Version 0.9.1)

Gephi is free open-source, leading visualization and exploration software for all kinds of networks and runs on Windows, Mac OS X, and Linux. It is our top preference as it is highly interactive and users can easily edit the node/edge shapes and colors to reveal hidden patterns. The aim of the tools is to assist users in pattern discovery and hypothesis making through efficient dynamic filtering and iterative visualization routines. As a generic tool, it is applicable to exploratory data analysis, link analysis, social network analysis, biological network analysis, and poster creation.

#### 2.1.1. Scalability

Gephi comes with a very fast rendering engine and sophisticated data structures for object handling, thus making it one of the most suitable tools for large-scale network visualization. It offers very highly appealing visualizations and, in a typical computer, it can easily render networks up to 300,000 nodes and 1,000,000 edges. Compared to other tools, it comes with a very efficient multithreading scheme, and thus users can perform multiple analyses simultaneously without suffering from panel “freezing” issues.

#### 2.1.2. Layouts

In large-scale network analysis, fast layout is a bottleneck as most sophisticated layout algorithms become CPU and memory greedy by requiring long running time to be completed. While Gephi comes with a great variety of layout algorithms, OpenOrd [26] and Yifan-Hu [27] force-directed algorithms are mostly recommended for large-scale network visualization. OpenOrd, for example, can scale up to over a million nodes in less than half an hour while Yifan-Hu is an ideal option to apply after the OpenOrd layout. Notably, Yifan-Hu layout can give aesthetically comparable views to the ones produced by the widely used but conservative and time-consuming Fruchterman and Reingold [28]. Other algorithms offered by Gephi are the circular, contraction, dual circle, random, MDS, Geo, Isometric, GraphViz, and Force atlas layouts. While most of them can run in an affordable running time, the combination of OpenOrd and Yifan-Hu seems to give the most appealing visualizations. Descent visualization is also offered by OpenOrd layout algorithm if a user stops the process when ~50–60% of the progress has been completed. Of course, efficient parametrization of any chosen layout algorithm will affect both the running time and the visual result.

#### 2.1.3. Postvisualization Analysis

Edge-bundling and famous clustering algorithms such as the MCL [29] do not come by default with Gephi but can be downloaded from Gephi's plugin library (~100 plugins). In addition, GeoLayout Gephi's plugin is very suitable to plot a network with geographical information. Coming to dynamic network visualization, Gephi is the forefront of innovation with dynamic graph analysis. Users can visualize how a network evolves over time by manipulating its embedded timeline. While visualization of a network over time is something very useful, its current algorithms are not suitable for large-scale networks. Similarly, for large-scale networks it is highly recommended for users to apply clustering algorithms using external command line applications and then import the clustering results to a visualization tool.

To study a network's topology, Gephi comes with a very basic but high quality network profiler showing basic statistics about the network such as the number of nodes, the number of edges, its density, its clustering coefficient, and other metrics. Automatically calculated node attributes such as node connectivity, clustering coefficient, betweenness centrality, or edge weight and similarly are trivial tasks and do not require long time to be calculated.

#### 2.1.4. Editing

Gephi is highly interactive and provides clever shortcuts to highlight communities, and shortest paths or relative distances of any node to a node of interest are offered. Moreover, users can easily adjust or interactively filter the shapes and colors of the network's edges and nodes according to their attributes in order to reveal hidden patterns. It is not the purpose of this review to tutor how to use such applications as this can be found in the tool's relevant help pages. While Gephi is a great option for large-scale network visualization, manual network importing, multiple network handling, and manual node/edge/label editing can be tricky as many options are well hidden in Gephi's user interface or supported by specific plugins.

#### 2.1.5. File Formats

Gephi can load networks in GEXF, GDF, GML, GraphML, Pajek (NET), GraphViz (DOT), CSV, UCINET (DL), Tulip (TPL), Netdraw (VNA), and Excel spreadsheets. Similarly, Gephi can export networks in JSON, CSV, Pajek (NET), GUESS (GDF), Gephi (GEFX), GML, and GraphML [30] files. The easiest way to talk with Cytoscape is through GraphML formats, with Tulip through GEFX files and with Pajek through NET files. Unfortunately, in its current version, communication with other tools through other common file formats such as JSON fails.

#### 2.1.6. Availability

**Figure 1:**
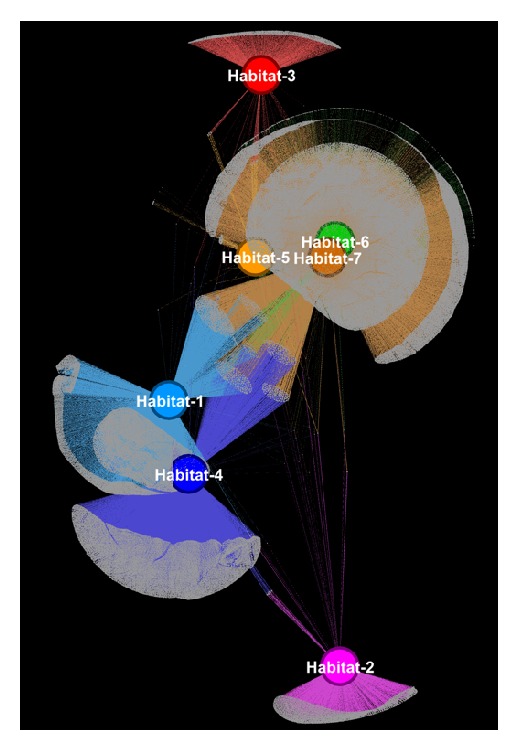
Gephi visualization of a network consisting of 202,424 nodes and 354,468 edges showing the distribution of 202,417 protein families across 7 habitats. A combination of OpenOrd and Yifan-Hu force-directed layout algorithm was used to calculate the node coordinates. Each habitat and its adjacent edges are colored uniquely. A step-by-step guide regarding the methods and the parametrization that were used is extensively described in the supplementary file.

Regardless of its very limited documentation, Gephi is a great, generic, nondedicated to biology, 2D network visualization tool. It mainly emphasizes fast and smooth rendering, fast layouting, efficient filtering, and interactive data exploration and we believe that it remains one of the best options for generic large-scale network visualization. A network example visualized by Gephi is shown in Figure 1. Gephi is available at: https://gephi.org/.

### 2.2. Tulip (Version 4.10.0)

Tulip is one of the easiest-to-use network visualization tools and a decent option for visualization of larger-scale networks. Due to its simplicity, it is highly recommended for nonexperts as it comes with an easy-to-use interface. It is written in C++ and enables the development of algorithms, visual encodings, interaction techniques, data models, and domain-specific visualizations. Compared to other tools, it offers very appealing visualizations especially after enabling its great edge-bundling algorithm.

#### 2.2.1. Scalability

In its current version, it is able to visualize thousands of nodes with hundreds of thousands of edges in an average computer and aims at becoming a great mediator between graph analysis and visualization. While Tulip is a top preference for medium-scale networks, to our experience it is not as scalable as Gephi.

#### 2.2.2. Layouts

Its great plethora of layout algorithms makes it one of the best options for graph layout. At the moment, it supports simple (circular, random), force-directed (i.e., Fruchterman and Reingold [28], Kamada and Kawai [31]), hierarchical, multilevel, planar, and tree layout algorithms, most of them optimized and implemented within the Open Graph Drawing Framework (OGDF) [32]. As opposed to the more conservative force-directed layout algorithms, Fast Multipole Multilevel Layout is highly recommended for large-scale networks. While its layouts are of a great quality, in order to save time, the strategy to first calculate the nodes layout with Gephi or Pajek and then import to Tulip is highly recommended.

#### 2.2.3. Postvisualization Analysis

By trying to bridge the gap between the analysis and visualization, Tulip comes with a rich pool of clustering and network topology analysis algorithms. Among others, Tulip currently implements the memory greedy but widely accepted greedy Markov Clustering (MCL) [29] as well as the fast and memory efficient Louvain Clustering [33] for unweighted graphs. In addition, Tulip incorporates various traditional algorithms for network exploration like algorithms to find the biconnected or the strongly connected components or algorithms dedicated to finding spanning trees or loops. Like before, for large-scale network analysis, running clustering algorithms externally is recommended.

In addition, Tulip comes with a very simple interface to ask topological questions. K-core decomposition of a graph, eccentricity centrality, degree, page rank, and betweenness centrality are few of the offered options and nodes' size or color can be adjusted according to a selected topological feature.

#### 2.2.4. Editing

While Tulip does not come with a great variety of predefined color schemes, users can manually change the color, the size, and the shape of any node, label, or edge and save and reload the status of a network. Unfortunately, it can process one network per session and users must be careful as sometimes the visualization and the editing panels do not coordinate. Unfortunately, simple tasks such as interactively selecting the in/out edges of a node directly from the visualization can take significant amount of time.

#### 2.2.5. Edge Bundling

While Tulip's renderer does not reach Gephi's or Cytoscape's resolution, it comes with one of the most appealing edge-bundling algorithms. Unfortunately, for large-scale network analysis, its edge-bundling algorithm can often become memory and CPU greedy so users must be patient. Finally saving the status of a bundled view compared to an unbundled view can lead to significantly higher storage requirements (see supplementary file for examples).

#### 2.2.6. File Formats

It accepts as input simple tab delimited, Pajek, GEFX, GML, GraphViz, JSON, TLPB, and UCINET files and exports to TLP, SVG, JSON, and GML formats. The easiest way to talk with Pajek is through NET files, with Cytoscape through GML or GraphML files, and with Gephi through GEFX files. Finally, Tulip comes with a very powerful generator of graphs of a user-defined size and topology.

#### 2.2.7. Availability

**Figure 2:**
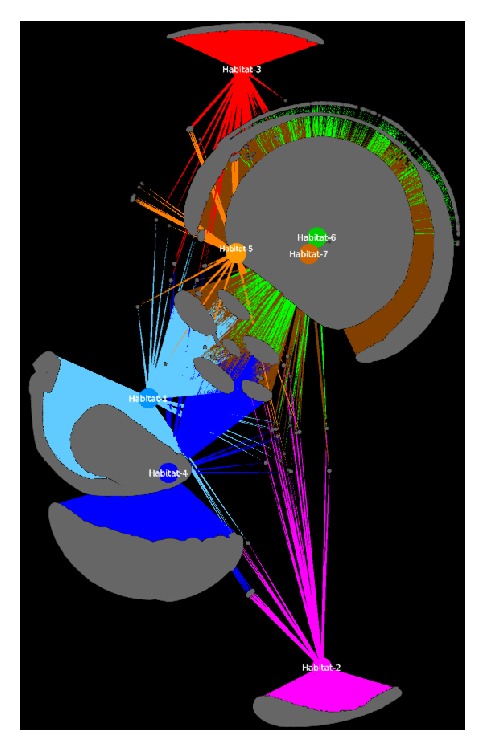
Tulip visualization of the same network like in Figure 1. The 7 habitats are highlighted and resized accordingly. An example of the same network after applying edge bundling is presented in the supplementary file. Nodes' coordinates were calculated using the Yifan-Hu layout algorithm from Gephi application.

Overall, Tulip is a generic 2D network visualization tool with a self-explanatory user interface and is suitable for large-scale node and edge layouting and analysis. A network example visualized by Tulip is shown in Figure 2. Tulip is available at: http://tulip.labri.fr/TulipDrupal/.

### 2.3. Cytoscape (Version 3.5.1)

Cytoscape open-source Java application is the most widely used 2D network visualization tool in biology and health sciences. It supports all kinds of networks (e.g., weighted unweighted, bipartite, directed, undirected, and multiedged) and comes with an enormous library of additional plugins (>250). It was initially implemented to analyze molecular interaction networks and biological pathways and was aiming at integrating these networks with annotations, gene expression profiles, and other state data. Although Cytoscape was originally designed for biorelated research, now it serves as a generic platform for complex network analysis and visualization by providing a basic set of features for data integration, analysis, and visualization.

#### 2.3.1. Scalability

Cytoscape implementations after version 3.0.0 come with tremendous rendering improvements, thus allowing Cytoscape to visualize large networks of hundred thousand nodes and edges. Despite these improvements, Cytoscape does not rank first for large-scale network analysis as it cannot scale significantly when it comes to analysis. Often Cytoscape's clustering and layout routines need great amount of memory and time. Therefore, for large-scale network analysis, it is suggested to run such processes in command line outside Cytoscape platform and load the results as node/edge attributes (groups in the case of clustering or coordinates in the case of a layout). In addition, Cytoscape is subject to Java's memory and running time limitations as most of its routines are implemented in Java.

#### 2.3.2. Layouts

Like other tools, it comes with a very rich variety of simple (grid, random, and circular) or more sophisticated (force-directed, hierarchical) layout algorithms. Notably, for large-scale network analysis, users must be careful and change the default layout algorithm before creating a view. A simple grid or a simple circular layout is recommended as Cytoscape's force-directed layouts are memory and CPU greedy and the application might “hang.” Another alternative could be OpenCL, one of the fastest layouts algorithms in Cytoscape. After version 3.2.0 OpenCL-based version is incorporated as a basic application. This layout is up to 100 times faster than the standard Prefuse layout and depends on the CyCL core app for OpenCL support. Nevertheless, calculating a first layout with Gephi or Pajek and then importing its results in Cytoscape can save time.

#### 2.3.3. Postvisualization Analysis

Cytoscape is the most successful tool for bridging the gap between analysis and visualization and it comes with a great plethora of layout, clustering, and topological network analysis algorithms. ClusterMaker plugin [34], for example, includes attribute cluster algorithms such as AutoSOME Clustering [35] and Eisen's hierarchical and *k*-Means clustering [36] as well as topology-based clustering algorithms such as affinity propagation [37], community clustering (GLay) [38], MCODE [39], MCL, SCPS (Spectral Clustering of Protein Sequences) [40], and transitivity clustering [41]. Most clustering results can be visualized as a newly constructed network preserving the original edges, or as a heatmap. Like before, for large-scale network analysis, users are encouraged to run such algorithms externally.

In addition, Cytoscape incorporates one of the most advanced network profilers to explore network topological features. Users are able to view simple statistics like the average connectivity, betweenness centrality, clustering coefficient, and others. While such calculations are trivial for large-scale networks, plotting a topological feature against any other could be slow.

Finally, Cytoscape's latest versions incorporate a rather useful but slow and memory inefficient edge-bundling algorithm, not recommended for large-scale analysis.

#### 2.3.4. Editing

Cytoscape is a protagonist in offering predefined visual styles and color schemes to create high quality and aesthetically beautiful visualizations. Its zooming and panning capabilities are very advanced and Cytoscape's satellite viewer makes it very easy for users to navigate and orient when the network is drawn outside the main canvas, something that is not trivial with Gephi. Finally, choosing adjacent nodes and edges from the UI is very responsive.

#### 2.3.5. File Formats

Cytoscape accepts many different input file formats such as its own CYS format, tab delimited, simple interaction file format (SIF), nested network format (NNF), graph markup language (GML), extensible graph markup and modelling language (XGMML), SBML [42], BioPAX [43], PSI-MI [44], GraphML, excel workbooks (.xls, .xlsx), and JSON. The easiest way to talk with Tulip and Gephi is through a GML format.

#### 2.3.6. Availability

**Figure 3:**
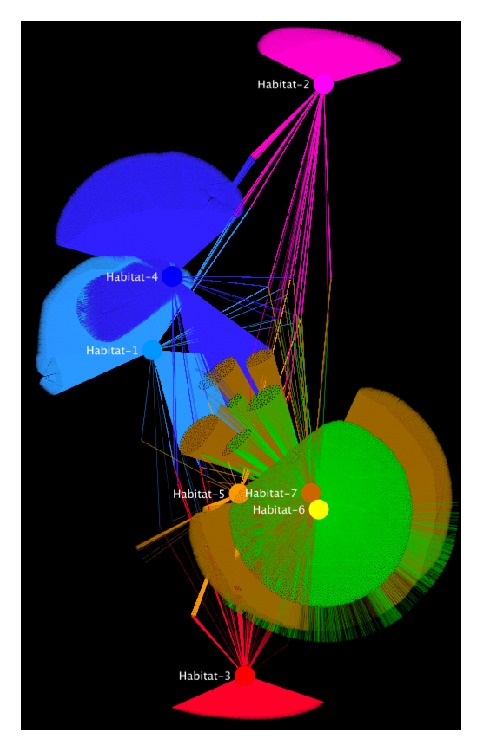
Cytoscape visualization of the same network like in Figure 1. The network consists of 202,424 nodes and 354,468 edges. The 7 habitats are colored accordingly. Like in Figure 2, coordinates were calculated using the Yifan-Hu layout algorithm from Gephi application.

Overall, Cytoscape is the best visualization tool today for biological network analyses. Despite its user friendliness, its rich documentation, and the tremendous improvement of its user interface after version 3.0, familiarity with the tool and its available plugins still requires a steep learning curve for more advanced tasks. Cytoscape store currently hosts more than 250 plugins, specifically designed to address and automate complicated biological analyses. Plugins for functional enrichment, Gene Ontology annotations [45], gene name mapping, integration with biological public repositories, efficient online data retrieval, pathway analysis, direct network comparisons, differential expression, and statistical analysis make Cytoscape unique of its kind and therefore today it currently is and expected to remain the number-one player for biological network analysis. A network visualized by Cytoscape is shown in Figure 3. Cytoscape is available at http://www.cytoscape.org/.

Finally, CytoscapeWeb [46] and Cytoscape.js are separate projects. They are two very strong efforts aiming to incorporate Cytoscape's main visual functionalities in browser-based applications, something that of course is not suitable for large-scale network analysis. Users can use Cytoscape and export the networks in JSON format for Cytoscape.js.

### 2.4. Pajek (Version 5.01)

Pajek is a generic, more than 20 years old, Microsoft Windows based network visualization tool, initially implemented for social network analysis, yet a very powerful application for analysis and visualization of massive networks.

#### 2.4.1. Scalability

Pajek can easily visualize million nodes with billion connections in an average computer by outperforming any other available tool in the field. Pajek-XXL is a special implementation of Pajek with emphasis on huge scale network analysis. It needs at least 2-3 times less physical memory than Pajek and most of Pajek's memory intensive operations are optimized to be much faster. The main philosophy of Pajek-XXL is to extract smaller but most interesting and informative parts of a larger network which can be further analyzed and visualized with more advanced tools. The highest possible number of vertices that Pajek64-XXL can handle has been increased to 2 billion as for ordinary Pajek the limit is 100 million. Pajek-XXL uses 32-bit (4 bytes) integers for vertices numbers. Thus, the highest number of vertices that Pajek-XXL can handle is set to two billion. If network contains more vertices Pajek-3XL must be used. Pajek-3XL uses 64-bit (8 bytes) integers for vertices numbers. The highest number of vertices that Pajek-3XL can handle is currently set to 10 billion but can easily be further increased. Notably, the space needed to store a network in Pajek-3XL and Pajek-XXL is exactly the same.

#### 2.4.2. Layouts

Graph layout, node merging, neighborhood detection, identification of strongly connected components, clique finding, manipulation of bipartite graphs, searching for shortest paths or maximum flows, clustering (i.e., Louvain), and computing centralities of vertices and centralizations of networks such as degree, closeness, betweenness, hubs and authorities, clustering coefficients, and Laplacian centrality are few of Pajek's capabilities. Notably, Pajek is memory efficient and very suitable for fast sparse network multiplication.

#### 2.4.3. File Format

Pajek accepts very strict file input formats. The easiest way to talk with Tulip and Gephi is through a .net file

**Figure 4:**
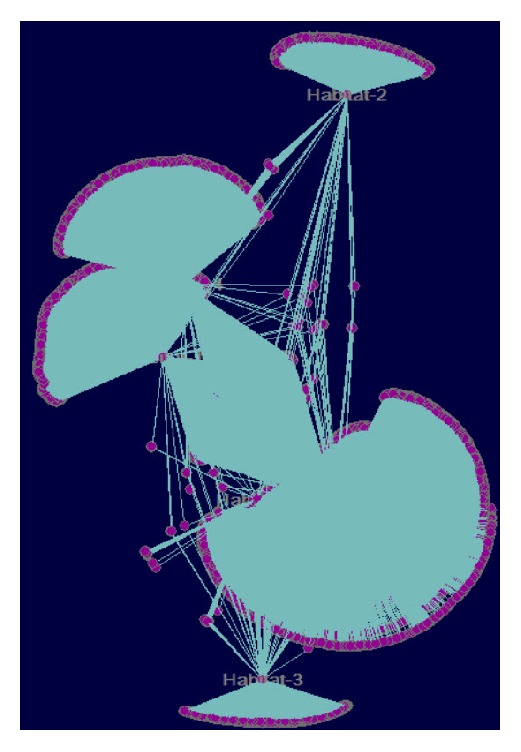
Pajek basic visualization of the same network like in Figure 1. The network consists of 202,424 nodes and 354,468 edges. Like in Figures 2 and 3, coordinates were calculated using the Yifan-Hu layout algorithm from Gephi application. Notably for a massive network it is highly recommended to first use Pajek layouting.

Pajek's user interface is simple, easy to get familiar with, and very responsive when it comes to analysis of massive networks. It was never intended to be the most advanced visualizer but it offers tremendous graph analyses methodologies thus making it a great candidate for analysis of massive networks and a great complement to the existing tools. A network example visualized by Pajek is shown in Figure 4. Pajek can be found at http://mrvar.fdv.uni-lj.si/pajek/.

## 3. Discussion

Despite the great plethora of available network visualization tools, due to the continuous increase of the data volume in health sciences, visualization and manipulation of large-scale networks with million nodes and edges still remain a bottleneck. While noninteractive libraries such as the Stanford Network Analysis Project (SNAP) [47], the outdated Large Graph Layout (LGL) [48], NetworkX [49], or the GraphViz [50] are preferred for backend calculations and large-scale static visualizations and while alternative network visualizations such as the ones offered by the Circos [51], HivePlots [52], or BioFabric [53] can partially solve the hairball effect, the implementation of user friendly interactive tools to handle and visualize such large graphs still remains a very complicated task. Therefore, for the purposes of this review article, we tested several available standalone applications and concluded that Pajek, Tulip Gephi, and Cytoscape are top candidates for large-scale network visualization and analysis.

In conclusion, while Cytoscape is the best and the most preferred tool for biological analyses, it has scalability and memory issues and therefore it is not our top pick for large-scale network visualization. On the contrary, we rank it first for biological analyses as it is accompanied by a great plethora of more than 200 plugins. Compared to Tulip, Gephi, and Pajek, it has the richest palette of predefined color styles, the most efficient collection of clustering algorithms, and the best network profiler for intranetwork comparison of topological features.

Gephi clearly outperforms Cytoscape in terms of scalability and memory efficiency and, in our opinion, it is the best generic visualization tool for layouting large-scale networks. While it is fairly straightforward to use, sometimes node/edge editing options are well hidden in its user interface thus making it a bit confusing for the user. On the other hand, Gephi offers very advanced visualizations by allowing users to perform multiple tasks simultaneously, something that is not always easy with Cytoscape or Tulip. Overall, we rank Gephi as first when it comes to balance between large-scale network visualization and basic analysis.

Tulip is our third best option for large-scale network visualization. Its best characteristics are (i) the edge-bundling layout and (ii) its simplicity in editing the node's/edge's colors, labels, and attributes. Tulip is highly recommended for beginners due to its self-explanatory user interface.

Finally, Pajek and Pajek-XXL are the most scalable tools and highly recommended for basic visualizations of massive networks with >10 billion nodes, network sizes that Cytoscape, Tulip, and Gephi cannot handle in their current versions. Unfortunately, the lack of operating system interoperability as well as the lack of input file format flexibility and the lack of appealing visualizations prevent Pajek from being the top tool for advanced visualizations.

All the aforementioned observations are summarized in Table 1. Even though they may vary from user to user depending on the expertise and the case study, in our opinion, Cytoscape, Tulip, Pajek, and Gephi still remain the best large-scale network visualization and analysis tools in systems and network biology.

## 4. Conclusion

It is unfair and not straightforward to directly compare visualization tools with each other as they are implemented to serve different purposes. Nevertheless, as biological network sizes increase over time, combining the complementary advantages from different tools is a good strategy. While several file formats to describe the structure of network have been standardized, our experience showed that many of them cannot be properly exported or imported across several tools. In addition, even in the best cases where such an import/export problem is absent, often node and edge attributes cannot be transferred. Therefore, we believe that a catholic network converted to accurately convert a file format into any other by simultaneously keeping the maximum information about the network's components is mandatory. This way, switching between tools and various visualizations will become easier and more straightforward.

## Supplementary Material

A step-by-step guide demonstrating how the networks in the figures can be generated.

## Figures and Tables

**Table 1:** Empirical evaluation of our top four interactive network visualization tools (Cytoscape, Gephi, Tulip, and Pajek) for large-scale biological network analysis.

	Cytoscape	Tulip	Gephi	Pajek
Scalability	*∗∗*	*∗*	*∗∗∗*	*∗∗∗∗*
User friendliness	*∗∗*	*∗∗∗∗*	*∗∗∗*	*∗*
Visual styles	*∗∗∗∗*	*∗∗*	*∗∗∗*	*∗*
Edge bundling	*∗∗∗*	*∗∗∗∗*	*∗∗*	—
Relevance to biology	*∗∗∗∗*	*∗∗*	*∗∗∗*	*∗*
Memory efficiency	*∗*	*∗∗*	*∗∗∗*	*∗∗∗∗*
Clustering	*∗∗∗∗*	*∗∗∗*	*∗*	*∗∗*
Manual node/edge editing	*∗∗∗*	*∗∗∗∗*	*∗∗∗*	*∗*
Layouts	*∗∗∗*	*∗∗*	*∗∗∗∗*	*∗*
Network profiling	*∗∗∗∗*	*∗∗*	*∗∗∗*	*∗*
File formats	*∗∗*	*∗∗∗*	*∗∗∗∗*	*∗*
Plugins	*∗∗∗∗*	*∗∗*	*∗∗∗*	*∗*
Stability	*∗∗∗*	*∗*	*∗∗∗∗*	*∗∗∗*
Speed	*∗∗*	*∗*	*∗∗∗*	*∗∗∗∗*
Documentation	*∗∗∗∗*	*∗*	*∗∗*	*∗∗∗*

*∗* = weaker; *∗∗* = medium; *∗∗∗* = good; *∗∗∗∗* = strongest.

## Additional Points

*Availability of Data and Materials*. The datasets used and/or analyzed during the current study are available from the corresponding author on reasonable request.
