# Supplementary material for: Empirical Comparison of Visualization Tools for Larger-Scale Network Analysis
Source: Adv Bioinformatics. 2017 Jul 18;2017:1278932. doi: 10.1155/2017/1278932 (PMC5540468; doi:10.1155/2017/1278932)
Supplement: Supplementary file 1 — A step-by-step guide demonstrating how the networks in the figures can be generated. [file 1278932.f1.docx]

**Supplementary File**

**Analyzing a large network with Gephi (v0.9.1), Tulip (v4.10.0), Cytoscape (v3.5.1) and Pajek (v5.01)**

In this supplementary file, we show a 20-step protocol on how to combine different tools towards a more appealing visualization of large-scale networks. In order to show representative visualizations generated by Gephi, Cytoscape, Tulip and Pajek tools, we constructed a graph consisting of 202,424 nodes and 354,468 edges showing the habitat distribution of 202,417 protein families across 7 habitats. Data were collected from the IMG integrated genome and metagenome comparative data analysis system whereas protein families originate from public metagenomes only.

Users are encouraged to start the analysis with the Gephi visualization tool to firstly set the layout of the network and then use Cytoscape for a deeper biological analysis or Tulip for a nicer edge bundling layout. For massive datasets, Pajek is preferable as a starting option, but it only runs on Windows and comes with a more complex to generate file format.

Step 1. Open Gephi 0.9.1 and import a tab delimited 2-column (unweighted) text file from *File > Import.*

Step 2. *Select file* from the path and define Tab as the delimiter.

Step 3. *Select agents*. Select from Source column to Target column.

Step 4. *Subfields in agents.* Users can leave the default options

Step 5. *Dynamic network.* Nothing to select here

Step 6. *Options*. Leave the default parameters

Step 7. *Finish*

Step 8. *Undirected* and *New graph*

Here users see the #of Nodes (202,424) and the #of edges (354,468)

Step 9. The network is then loaded and appears in the form of grid layout.


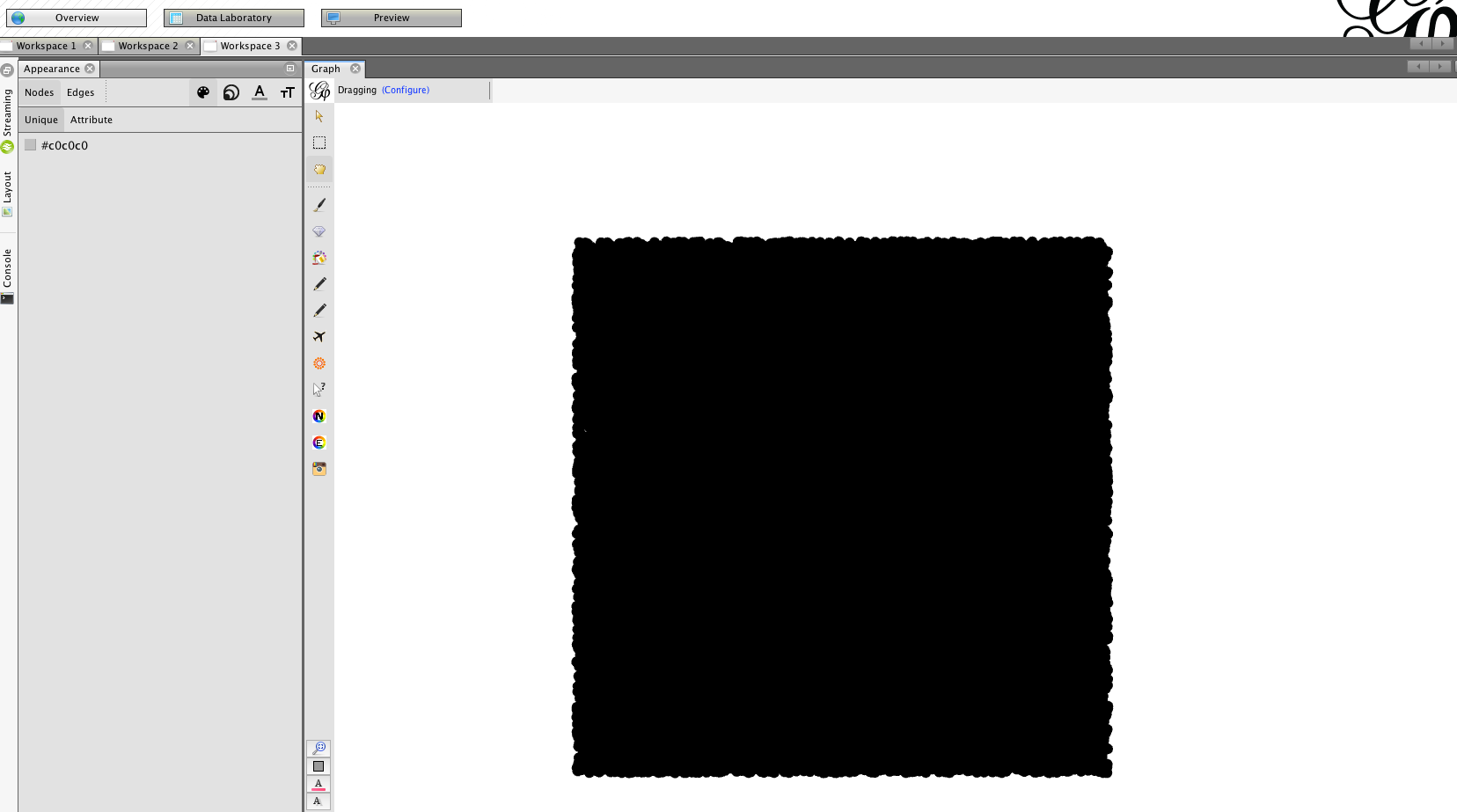


Step 10. OpenOrd is a fast spring-embedded layout algorithm which creates tight clusters. Users can go to *Window > Choose layout* and select OpenOrd. OpenOrd is highly recommended for very large networks due to its speed. OpenOrd can be stopped anytime while it runs. It can give a very descent visualization even when ~55-65% of its progress is completed. After this point, it tends to create visualization of tighter clusters. For the default parameters (Liquid 25%, Expansion 25%, Cooldown 25%, Crunch 10%, Simmer 15%, Edge cut 0.8, Num threads 39, Num iterations 750, Fixed Time 0.2 and a random seed), after ~50 minutes calculation time in a 2.8 GHz Intel Core i7, 16GB RAM Mac machine it will generate a network visualization like the one presented below:


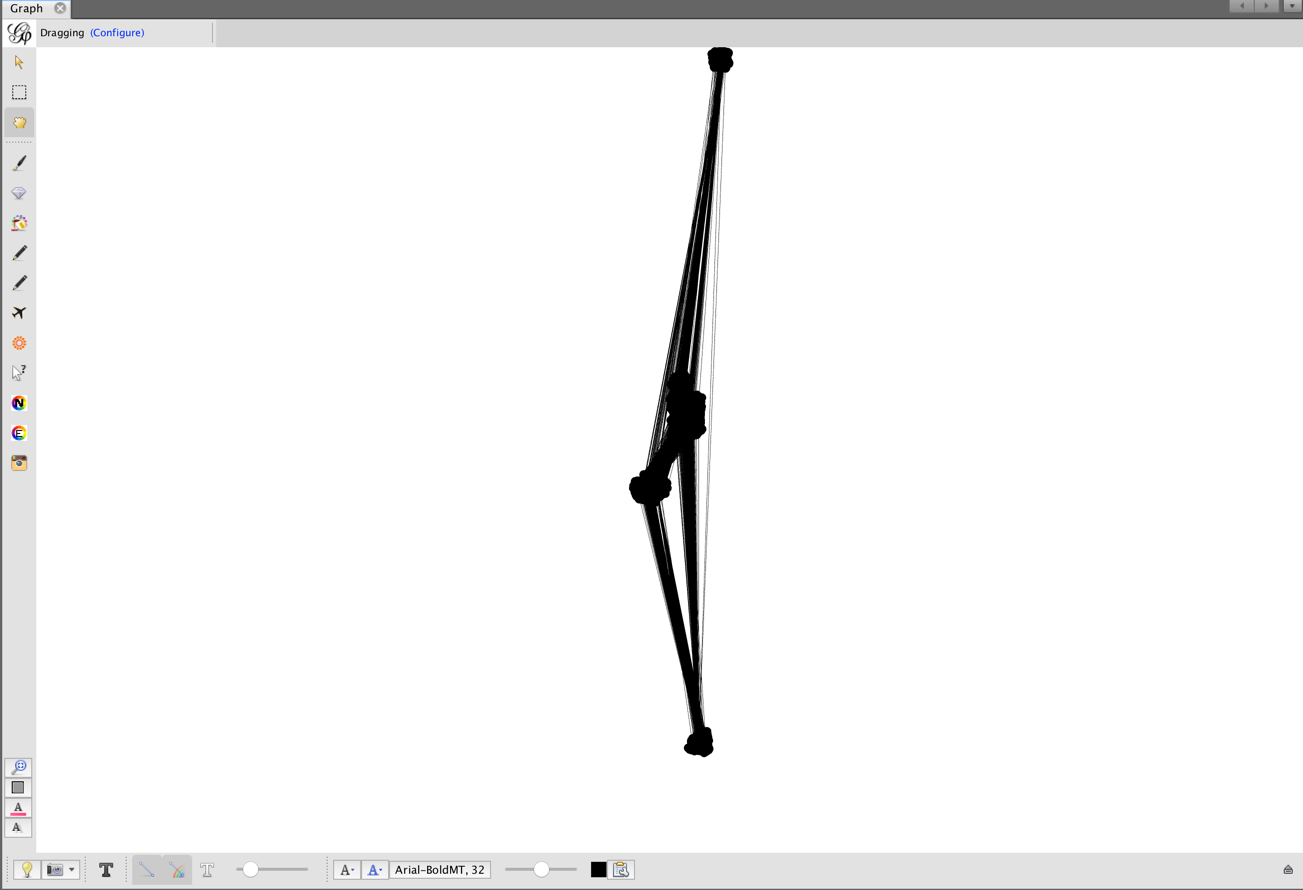


Step 11. For a more aesthetic visualization, users can utilize the Yifan-Hu layout algorithm. While there is always the option to run Yifan-Hu directly, it is highly recommended to first run the OpenOrd algorithm. Yifan-Hu will take into account the node coordinates calculated by OpenOrd and will continue from that point. After OpenOrd’s first coordinate calculations, Yifan-Hu will run much faster and will give a network closer to a Fruchterman-Reingold view. While OpenOrd tends to make clusters very tight, Yifan-Hu spreads them further in order to give a more aesthetic visualization. Notably, if users decide at any time to pause OpenOrd and rerun it, calculations will start from scratch, something that does not happen with Yifan-Hu.

To run the algorithm, users can go to: *Choose layout* and select Yifan-Hu. For the default parameters (Liquid 25%, Expansion 25%, Cooldown 25%, Crunch 10%, Simmer 15%, Edge cut 0.8, Num threads 39, Num iterations 750, Fixed Time 0.2) and after ~30 minutes calculation time in a 2.8 GHz Intel Core i7, 16GB RAM Mac machine it will generate a visualization like the one below:


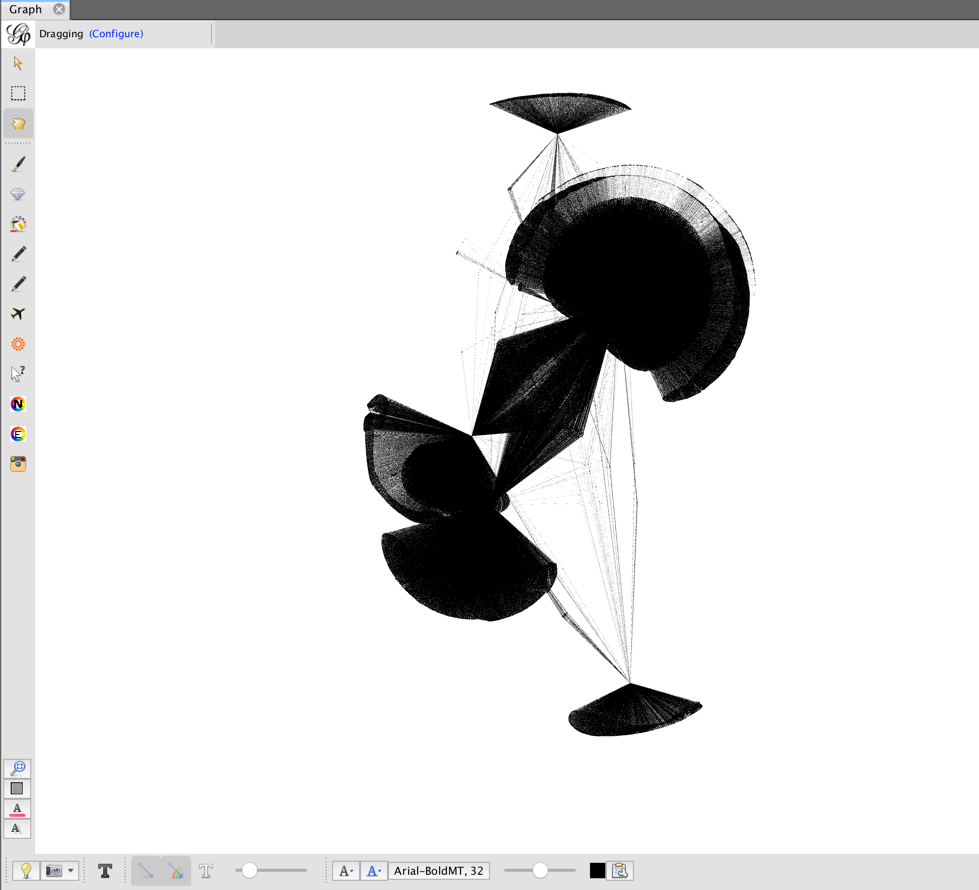


Step 12. Edit the color scheme. From the *Data Laboratory Tab*, users can edit the labels and the node color and size. Similarly, from the *Graph Tab* users can change the color background. The network will look like below:


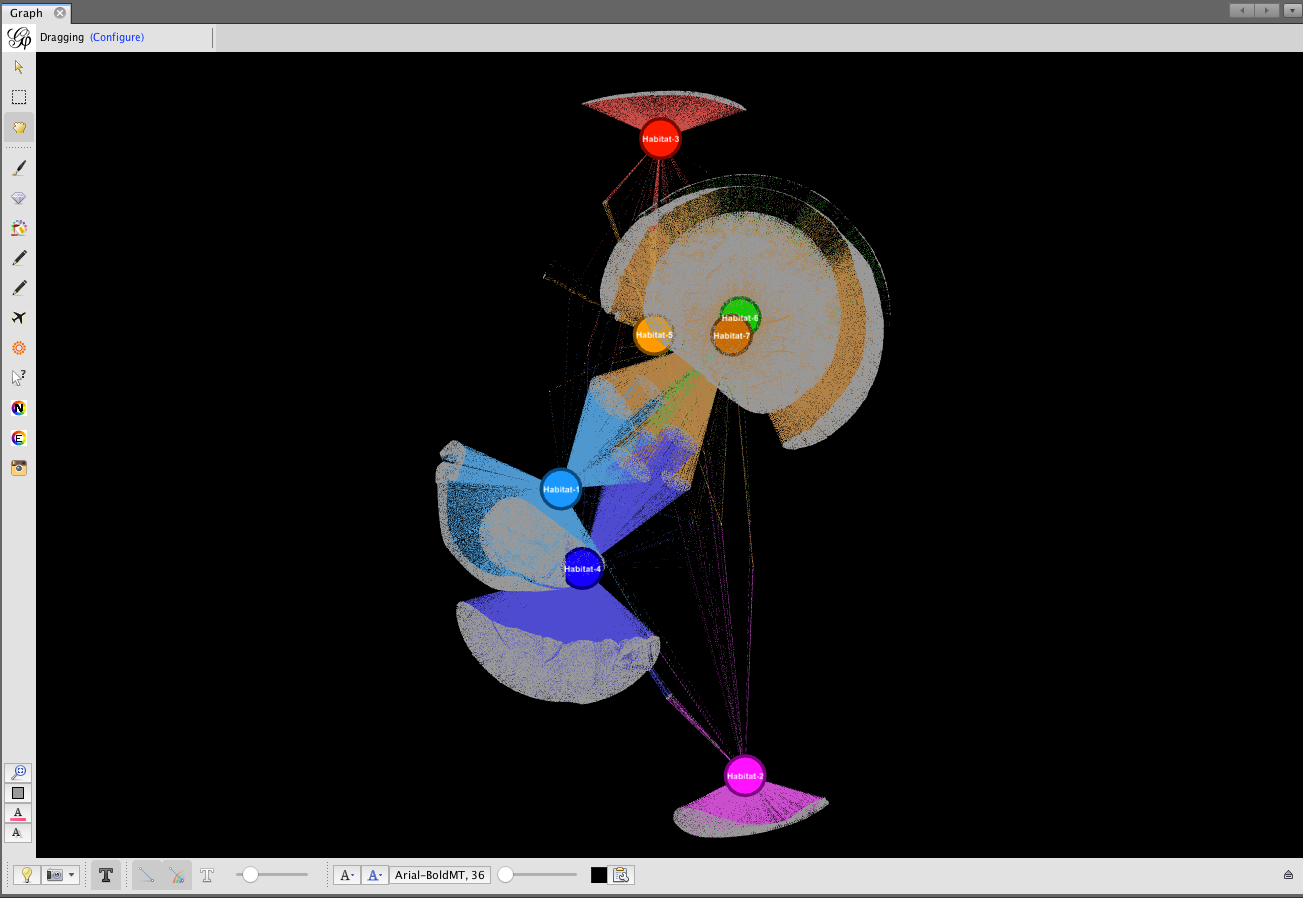


Step 13. Export to Tulip

File > Export > Graph File > GEFX

Step 14. Import from Tulip (v 4.10.0)

File > Import > GEFX

As color edges cannot be transferred, initially the network will look like the one below:


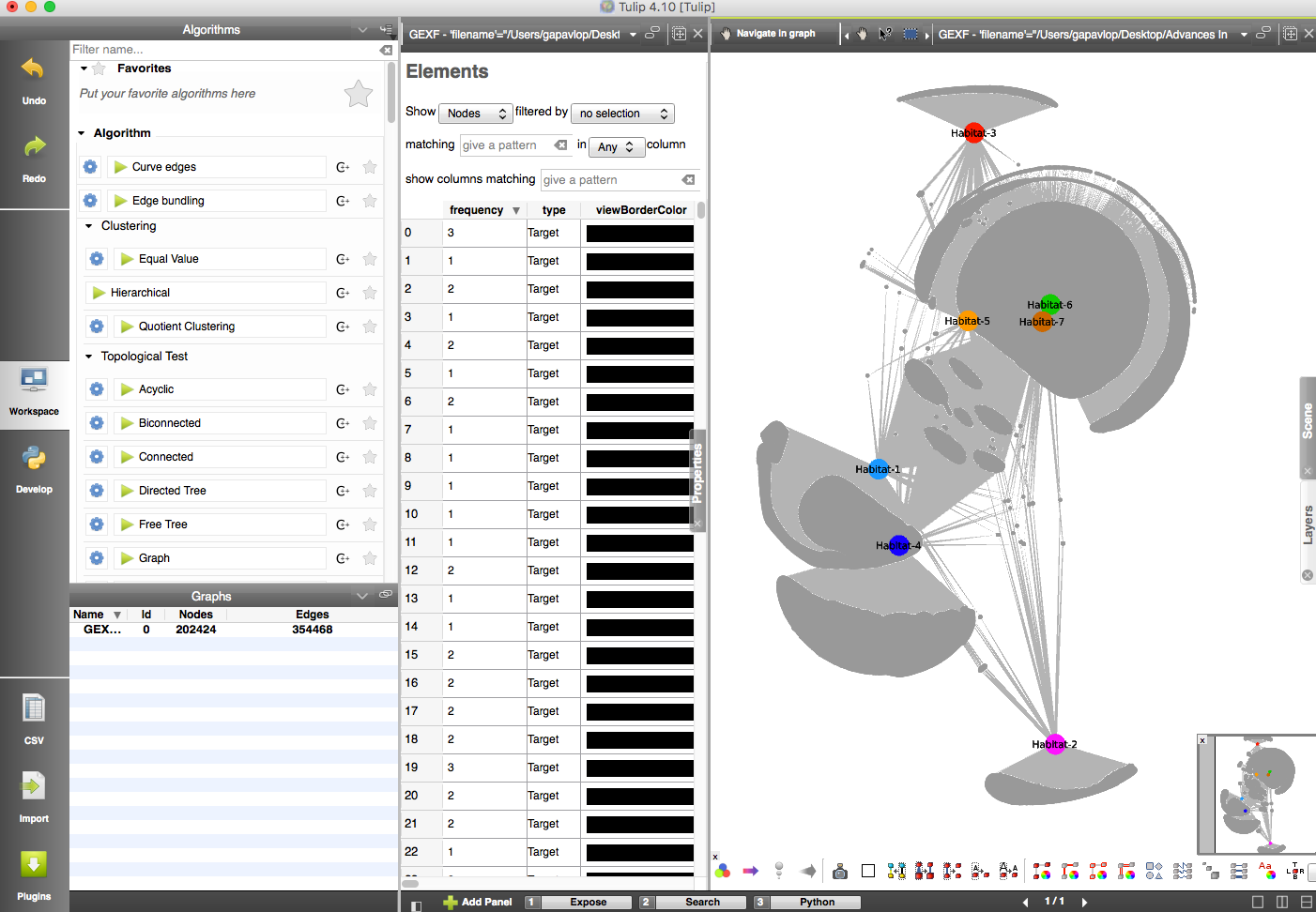


Step 15. Users can manually adjust colors of nodes and edges with Tulip (v 4.10.0)

Right click on a node and select *Toggle Selection of output Edges*. Once the output edges are highlighted (time consuming process), users can go to the *Elements Tab* and select: *Show edges filtered by viewSelection*. When the list gets filtered, then users can select all edges (ctrl+a) and right click on a color from the *ViewColor* column and then select *Set Values of Selected Edges of the Current graph*.

The visualization will become:


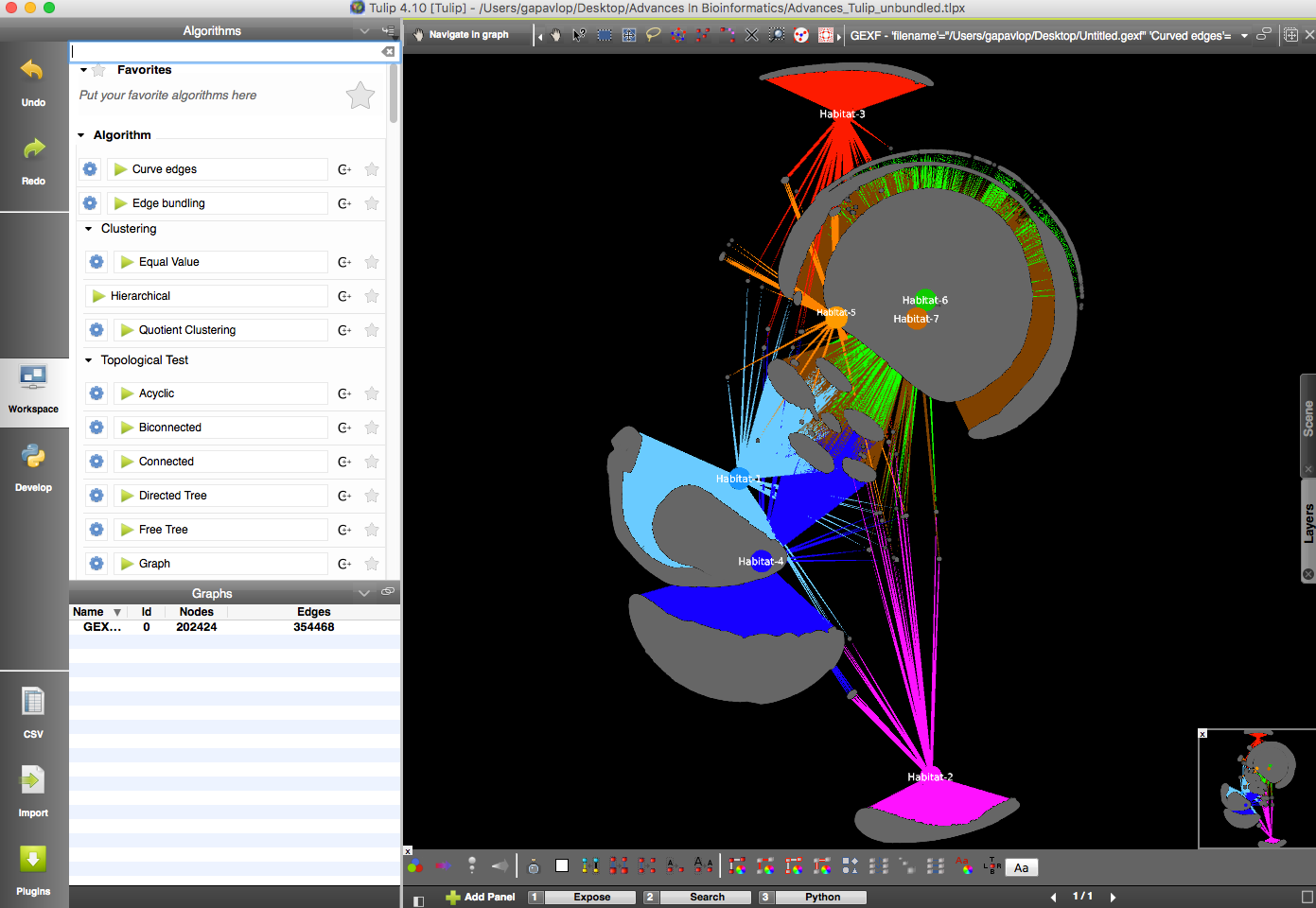


At this point users are encouraged to save the visualization in Tulip’s standard format.

For very dense networks, edge bundling will often give a much more appealing visualization. To apply edge bundling first users can optionally go to the *Algorithms Panel* and Run the *Fast Overlap Removal* algorithm to first make sure that nodes do not overlap. Then users can run the edge bundling algorithms from Tulip’s *Algorithms Panel*.

The network will look like the one below:


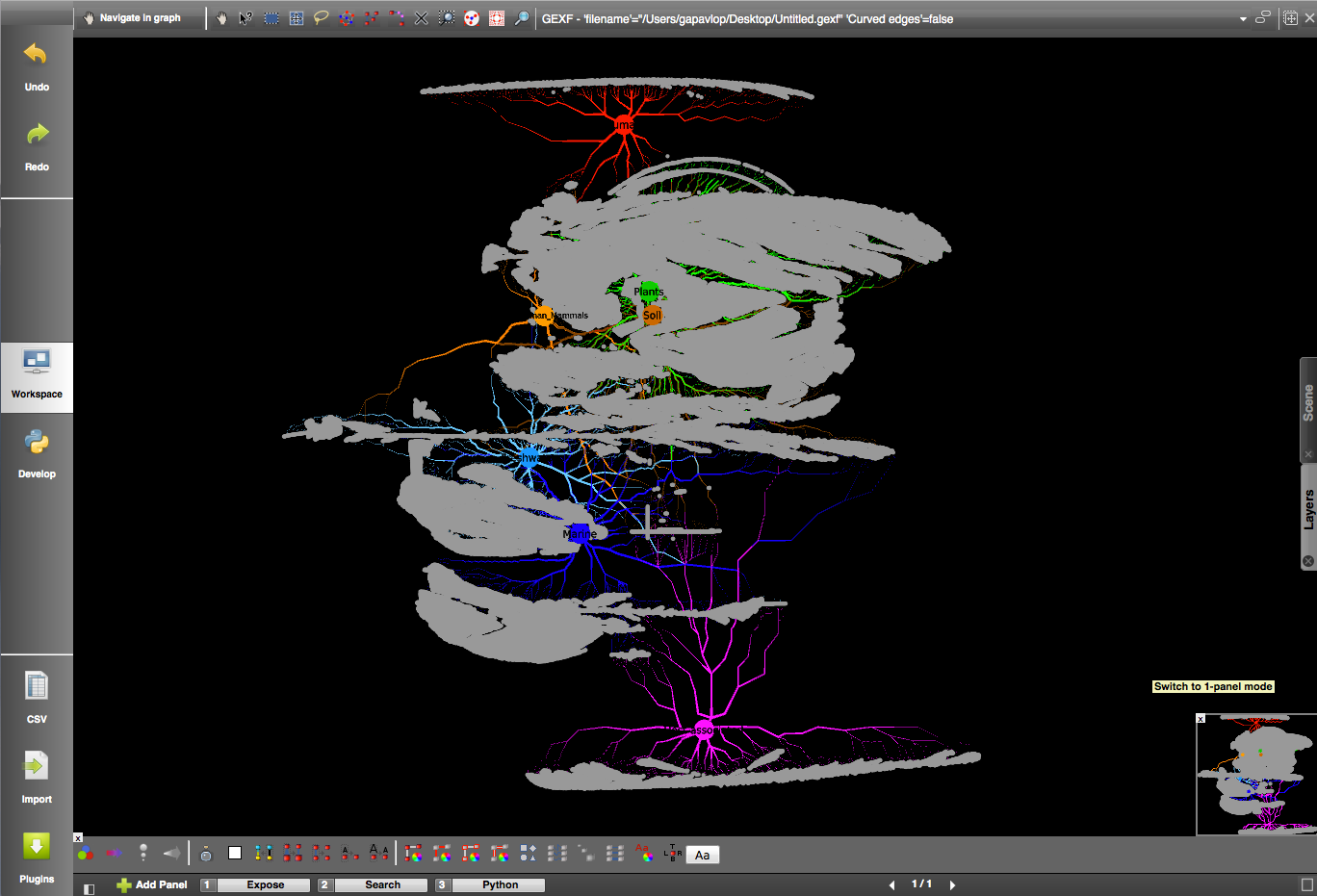


Caution!

- Saving the network in this form can significantly cause storage problems as the generated file is many orders of magnitude larger than the original one. In this example, the unbundled colored network has size ~7MB whereas the bundled version of the same network ~420MB.
- Edge bundling is a very time-consuming process. For the default parameters (long edges 0.9, split ratio 10, iterations 2, max thread 0, edge node overlap: false, sphere layout: false, 3D layout: false, grid graph: false and size: ViewSize), a ~3-hour calculation time was necessary for this network.
- Edge bundling can be applied directly to a loaded network without first running the Fast Overlap Removal algorithm. In this scenario, in rare cases, Edge bundling might crash when node coordinates overlap. To avoid this crash, running the Fast Overlap Removal algorithm (fast process) first is recommended always at the cost of changing the initial layout which might lead to a less appealing node positioning.

Step 16. Users can export a GML file from Tulip to Cytoscape. To do that, users must go to the *Graphs panel*, select the network of preference and right click on the network name. Then, an export popup window will appear and users can define the path where the GML file will be stored to.

Step 17. To load the GML file from Cytoscape, users must first open Cytoscape and go to *File > Import* and then select the GML file. Once the network is loaded, edge colors will be ignored and the network will look like the one below:


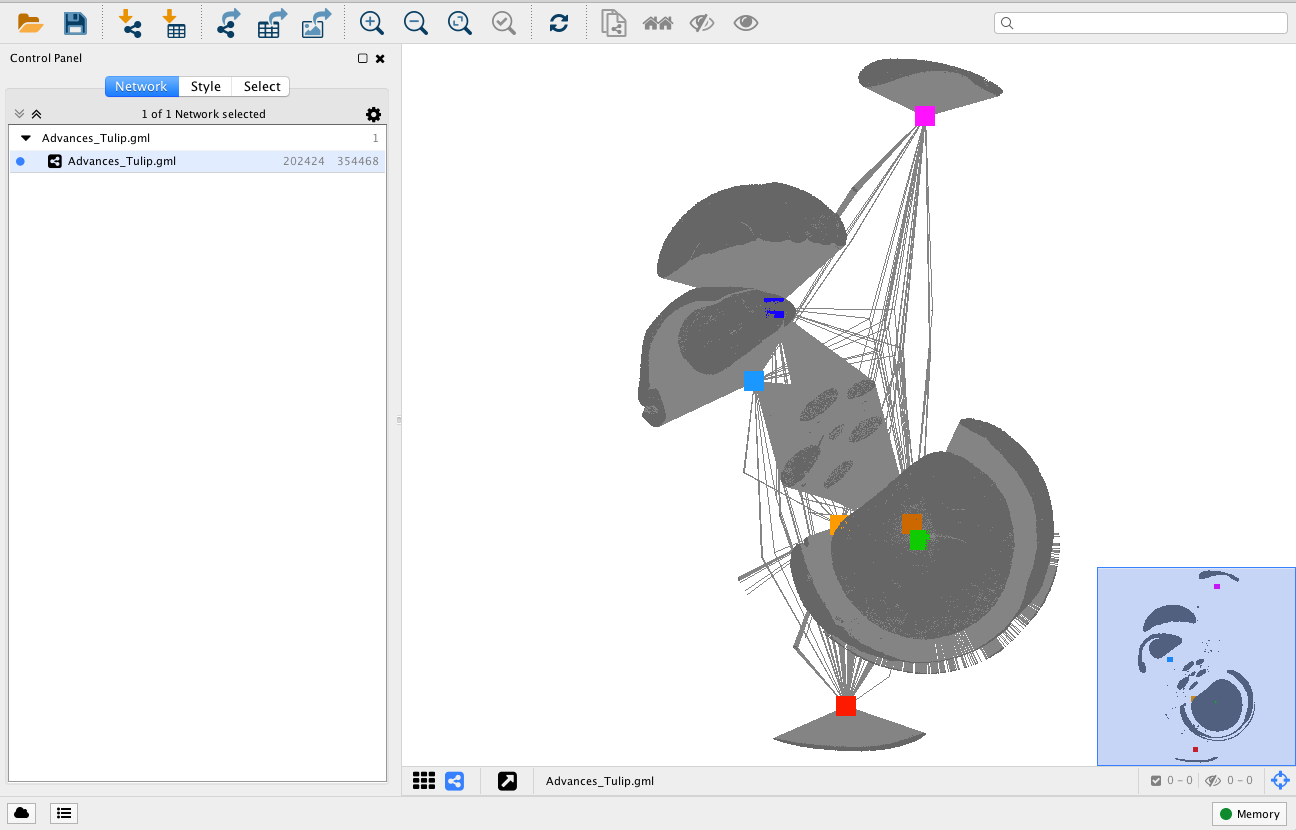


To edit and color the network please follow the steps:

1. Edit the GML file with a text editor and set each node’s type to “circle”
2. Go to Cytoscape and then *View>Show* Graphics details
3. To change the edges of the nodes You first needs to assign an attribute for each edge. As here we have 7 habitats, users to define a habitat for each edge (see interaction column).
4. To do that first go to the top right search box and type for example “Marine*”. This will select all of the edges containing the word “Marine”. Then go to the Table Panel (*View>Table* panel) and right click and edit the first cell of a newly created column (attribute column). Once you edit the first cell right click again and choose *Apply to selected edges*. After, this all the selected edges will have the attribute “Marine”
5. When you do that for each habitat then you can go to the *Edge Tab of the Control Panel* and edit the *Stroke Color* property field. Choose *Discrete Mapping* and select a color for each Habitat (attribute). Then let Cytoscape refresh the visualization.

Once all these steps are done iteratively, the visualization should look like below:


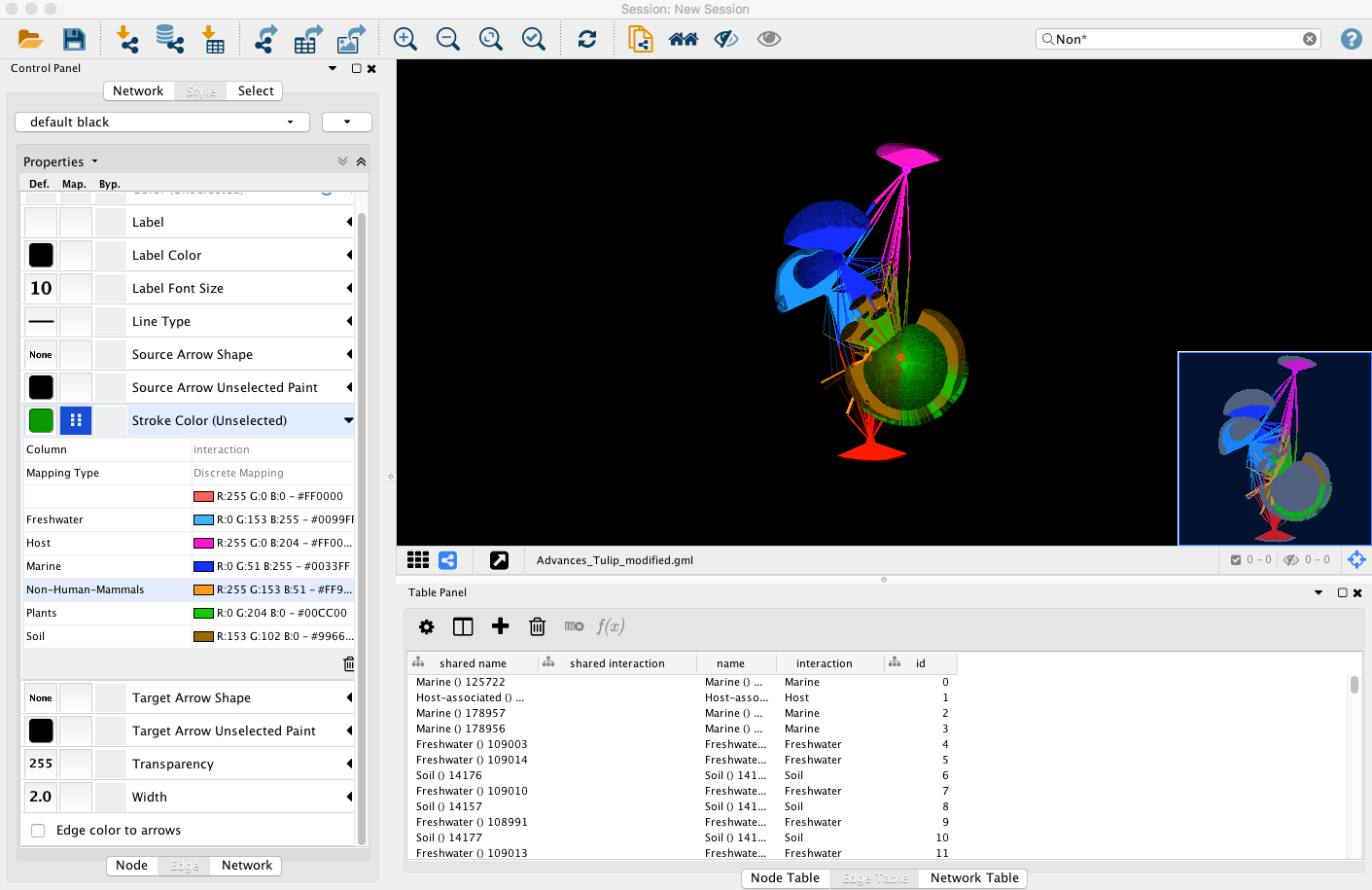


Step 18. To make a network Pajek-readable, like with Tulip, users can export a .net file from Gephi application (*File > Export > Graph File > .net*).

Step 19. To load the network in Pajek, users can firstly open Pajek application and import the .net file from *File > Network > Read* menu.

Step 20. From the draw menu (*Draw> Network* and then *Options > Transform > fit area > max(x,y,z))* users can create a first drawing of the network which looks like the one below:


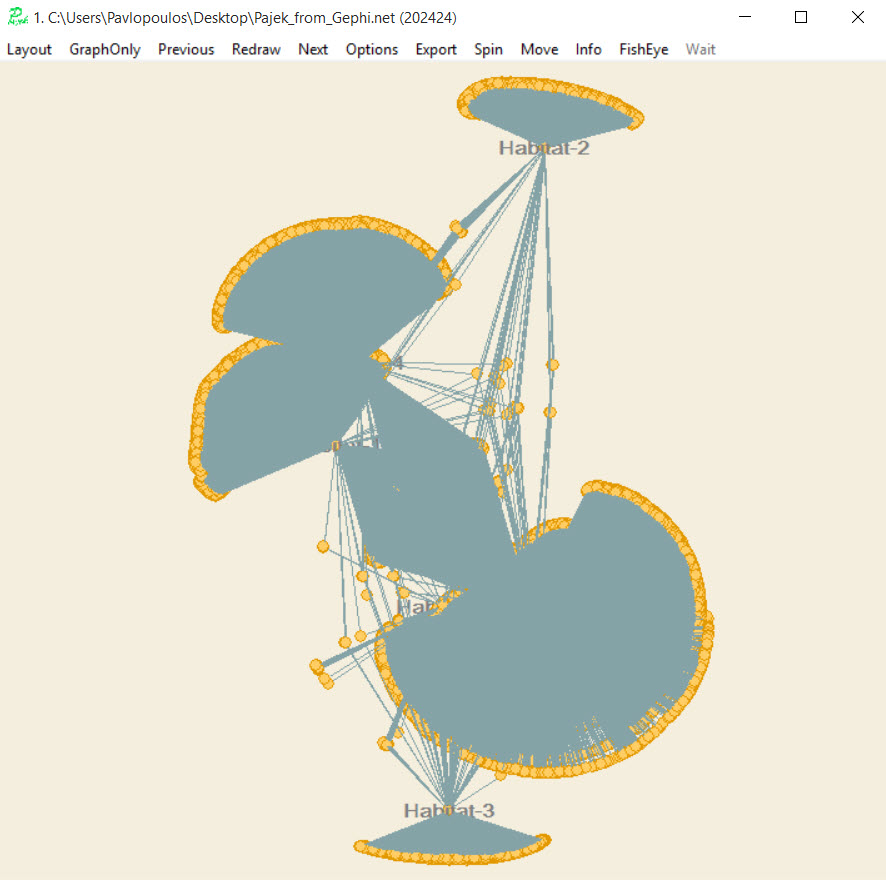


To change the node and edge shapes and colors users are encouraged to directly edit the input file.

The .NET file format is explained here: <http://vlado.fmf.uni-lj.si/pub/networks/pajek/svganim/1.10.7.1/PajekToSvgAnim.pdf>

If Pajek complains about the number of vertices, users can increase the default parameter (100,000) from *Options > Read-Write* menu.

Concluding Note

- Unfortunately, file converters are semi-complete in these versions of the tools. Thus, one would expect that using a generic network format like JSON would be a very good strategy to visualize a network with various tools. Our experience shows that this is not happening, as the tools most of the times fail to properly load or export such file formats. For example, exporting a network in GML format is good enough to be imported to Cytoscape, but reading a GML format exported by Gephi with Cytoscape fails. Additionally, Cytoscape can efficiently read GraphML files, but every number is stored as a separate attribute/column (e.g. R, G, B colors and x, y coordinates). Often using various file formats to transfer all node and edge attributes fails as colors and other properties for example are not transferable.

Below we give a schematic representation of the file formats that we used to make tools communicate with each other. Notably, there are more combinations than the ones showed here like for example communication through JSON format which unfortunately fails.


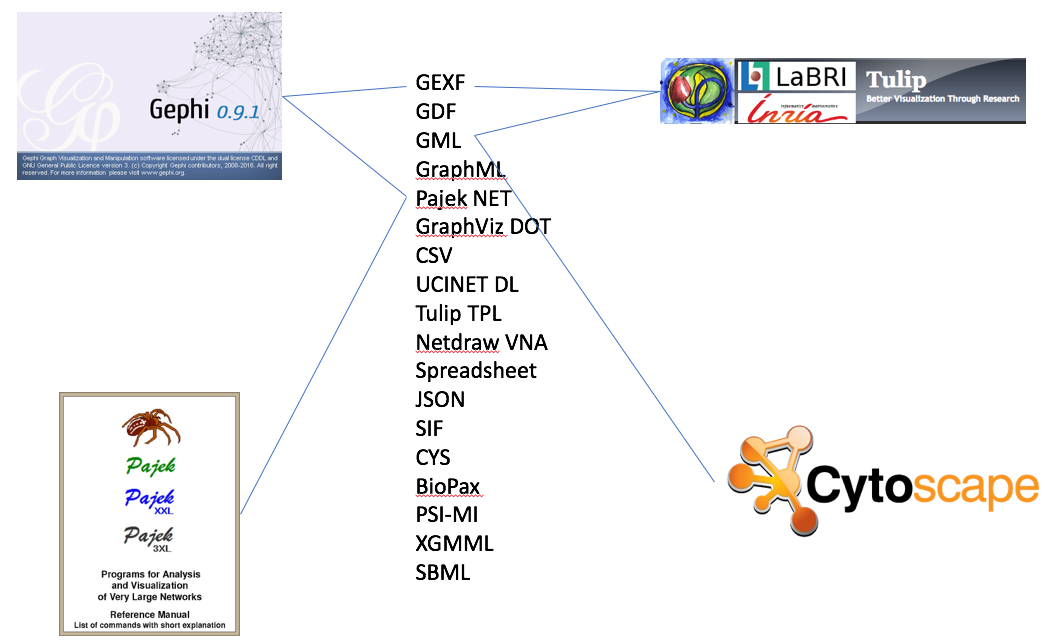


Suggestion

- An efficient converter to properly transform a network file from one format to another is mandatory in order to take advantage of the full capabilities of each tool.
